# Microglia Development and Maturation and Its Implications for Induction of Microglia-Like Cells from Human iPSCs

**DOI:** 10.3390/ijms22063088

**Published:** 2021-03-17

**Authors:** Johannes Wurm, Henna Konttinen, Christian Andressen, Tarja Malm, Björn Spittau

**Affiliations:** 1 Anatomy and Cell Biology, Medical School OWL, Bielefeld University, 33615 Bielefeld, Germany; johannes.wurm@uni-bielefeld.de (J.W.); christian.andressen@uni-bielefeld.de (C.A.); 2 Neuroinflammation Research Group, A.I. Virtanen Institute for Molecular Sciences, University of Eastern Finland, 70211 Kuopio, Finland; henna.konttinen@uef.fi (H.K.); tarja.malm@uef.fi (T.M.)

**Keywords:** microglia maturation, TGFβ, iPSC, microglia-like cells, hiMGLs

## Abstract

Microglia are resident immune cells of the central nervous system and play critical roles during the development, homeostasis, and pathologies of the brain. Originated from yolk sac erythromyeloid progenitors, microglia immigrate into the embryonic brain parenchyma to undergo final postnatal differentiation and maturation driven by distinct chemokines, cytokines, and growth factors. Among them, TGFβ1 is an important regulator of microglial functions, mediating homeostasis, anti-inflammation, and triggering the expression of microglial homeostatic signature genes. Since microglia studies are mainly based on rodent cells and the isolation of homeostatic microglia from human tissue is challenging, human-induced pluripotent stem cells have been successfully differentiated into microglia-like cells recently. However, employed differentiation protocols strongly vary regarding used cytokines and growth factors, culture conditions, time span, and cell yield. Moreover, the incomplete differentiation of human microglia can hamper the similarity to primary human microglia and dramatically influence the outcome of follow-up studies with these differentiated cells. This review summarizes the current knowledge of the molecular mechanisms driving rodent microglia differentiation in vivo, further compares published differentiation protocols, and highlights the potential of TGFβ as an essential maturation factor.

## 1. Introduction

Microglia are a specialized subset of myeloid cells and represent the resident immune cell population of the central nervous system (CNS). Recent achievements in deciphering the development and differentiation of microglia have resulted in various protocols to generate human microglia-like cells (hiMGLs) from human-induced pluripotent stem cells (hiPSCs) in vitro. Although these approaches result in hiMGLs that—at least partially—share the molecular signatures with human microglia in vivo, available differentiation protocols should consider recent findings elucidating microglia development and maturation in vivo. Here, we summarize the mechanisms of microglia differentiation in mice and compare various protocols using hiPSCs to generate hiMGLs in vitro. Special focus is given on the role of transforming growth factor β1 (TGFβ1), its essential functions during microglia maturation, and its potential as an indispensable factor for the generation hiMGLs. The maturation of these cells closely mimicking resident human microglia is a prerequisite for future functional studies and may also pave the way for future microglia replacement strategies in humans.

## 2. Mouse Microglia Development and Maturation In Vivo

### 2.1. Mouse Microglia Origin and Development

**Figure 1:**
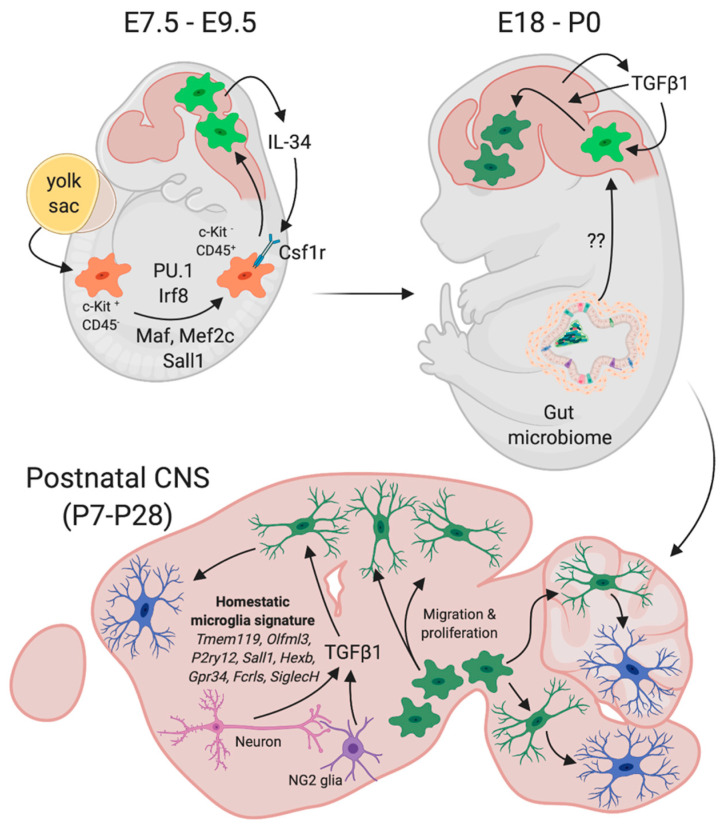
Molecular mechanisms underlying mouse microglia development and maturation in vivo. The figure was created using BioRender. Available online: https://biorender.com (accessed on 17 March 2021).

In contrast to other macrophage populations, microglia and CNS-associated resident macrophages (border-associated macrophages (BAMs)) originate from primitive macrophages at the first wave of hematopoiesis (for an overview of mouse microglia development and maturation, see Figure 1) [1]. These cells develop from early erythro-myeloid progenitors (EMPs) in the yolk sac at embryonic day E8.5 in a PU.1- and Irf8-dependent manner [2]. Unlike monocyte-derived macrophages, these primitive macrophages do not require the transcription factor Myb for their development [3]. They initially express the receptor tyrosine kinase c-Kit and lack the expression of CD45 and gradually lose c-Kit expression, while increasing the expression of CD45 during their further maturation [1,4]. Overlapping with the establishment of the primary blood circulation system, primitive macrophages start to migrate towards the neural tube to populate the developing brain and the spinal cord around E9.5, where they finally give rise to microglia [1,3]. This crucial migration step is dependent on the neuronal expression and secretion of Interleukin 34 (IL-34), which is sensed by the colony stimulating factor-1 receptor (Csf1r) being expressed by EMPs and developing microglia [1,5]. Moreover, microglial Csf1r signaling is further essential to mediate the survival of adult microglia [6]. In addition to the above-mentioned factors, the signal-dependent transcription factors Maf, Mef2c, and Sall1 are critical for proper microglia development [7,8,9].

Along with the establishment of the blood–brain-barrier (BBB) prenatally and after birth, the microglia population increases in the first postnatal weeks due to intraparenchymal proliferation and is further maintained in the adult CNS by a constant balance between microglial proliferation and apoptosis [10,11]. Under healthy physiological conditions, there is virtually no contribution of progenitors and/or monocytes recruited from peripheral blood circulation to maintain and support stable microglia numbers [12,13]. The gut microbiome, as well as the interaction of maturing microglia with various cell types within distinct developing CNS regions during the first postnatal weeks, is critical for the establishment of a region-specific microglia heterogeneity [14,15].

### 2.2. Mouse Postnatal Microglia Maturation

After prenatal microglial colonization of the brain parenchyma and subsequent pre- and postnatal microglial proliferation, the maturation process of these cells is further characterized by the induction and establishment of a microglia-specific gene expression pattern that distinguishes them from other macrophage populations [16,17,18,19,20,21]. These microglia-enriched genes are referred to as microglia homeostatic markers and are expressed by adult microglia under physiological conditions throughout different brain regions [22]. The expression of these genes, including *Tmem119*, *Olfml3*, *P2yr12*, *Sall1*, *Hexb*, *Gpr34*, *Fcrls*, or *SiglecH*, is upregulated within the first two postnatal weeks in mice and correlates with the activation of TGFβ signaling in microglia [18]. Immunohistochemistry and transgenic approaches have suggested neuron-derived TGFβ1 and TGFβ2 released by NG2-glia to be critically involved in triggering postnatal microglia maturation [18,23]. Further studies have clearly demonstrated that TGFβ1 is essential for microglia development [24] and maturation [25]. The deletion of TGFβ1 expression in the CNS [17], impairment of extracellular TGFβ1 processing and activation [26,27], or silencing of microglial TGFβ1 signaling by deletion of the TGFβ1 receptor *Tgfbr2* [28] resulted in a loss of microglia maturation, characterized by a lack of homeostatic microglia marker expression. Moreover, affected microglia further display an inflammatory phenotype, as evidenced by the increased expression of *ApoE*, *Axl*, *Cybb*, *Cd74*, *H2-Aa*, or *Il1b*, further emphasizing the importance of microglial TGFβ signaling to regulate microglia reactivity [28,29]. Although the functional significance of most of the homeostatic microglia markers is not well understood, the expression of these microglia-enriched genes strongly suggests their importance for mediating microglia functions, especially under physiological conditions [22,25]. This hypothesis is further supported by recent reports demonstrating the expression of the purinergic receptor *P2ry12* to be essential to promote microglial-driven neuroprotection and to support neuronal functions by dynamic interactions with synapses, as well as the microglia-mediated maintenance of BBB integrity after cerebrovascular damage [30,31]. Although TGFβ1 and microglial TGFβ signaling have been well established as crucial drivers of postnatal microglia maturation, it is likely that further molecular and cellular cues might be involved in final microglia differentiation and maturation processes in mice and humans. In addition, recent reports have described astrocyte-derived IL33 and the presence of CD4^+^ T cells in the CNS to be of critical importance for postnatal microglia maturation [32,33]. These recent studies indicate the molecular complexity involved in microglia maturation and further underpin the importance of future studies aiming to elucidate these mechanisms controlling the terminal developmental process.

### 2.3. Functional Consequences of Impaired Microglial Maturation

Even though it is evident that microglia play a role in various CNS pathologies [22,34], although their exact role is not established in disease progression, still, their functions during CNS development and maintenance under physiological conditions remain unclear and is a major challenge in the microglia field. To date, microglia have been demonstrated to play important roles during pre- and postnatal CNS development by performing the synaptic pruning and active control of neuronal circuit formation [35,36,37], support of neuron survival and oligodendrocyte-mediated postnatal myelination [38,39], and promotion of learning-dependent synapse formation and maintenance of synapse function and integrity [40,41]. It is likely that these processes only represent a part of the full functional repertoire of microglia and that future studies will shed light on additional essential microglia functions in health. Furthermore, the above-mentioned recently found microglia roles suggest that impaired microglial maturation could also have detrimental consequences for postnatal CNS development.

Indeed, the loss of microglial TGFβ signaling has been described to result in the development of spastic motor deficits as a consequence of impaired postnatal myelination of grey and white matter tracts, which is caused by disturbed oligodendrocyte maturation [28]. Moreover, in the presence of immature microglia, a substantial loss of cortical inhibitory interneurons has been demonstrated [28]. Supporting the hypothesis that the impairment of microglial maturation has detrimental effects on CNS development, the postnatal depletion of microglia did not result in the phenotypes observed in mice with immature microglia [28]. Taken together, these results underline the importance of the establishment of the homeostatic microglia-enriched gene expression signature for CNS development and postnatal maturation. This further suggests that a full microglia maturation phenotype needs to be induced in microglia-like cells generated from human iPSCs for subsequent functional analyses and/or replacement studies to better mimic the full spectrum of in vivo microglia.

## 3. Generation of Human Microglia-Like Cells (hiMGLs) from Human-Induced Pluripotent Stem Cells (hiPSCs)

The current knowledge about microglia development is mainly based on experimental animal studies performed in rodents with minor support from a few studies of aborted human embryos [42,43]. Although human and rodent microglia share most of their key functions and expression profiles, differences at a regulatory level have been identified. Pathways primarily involved in the modulation of microglial immune responses, such as the TGFβ-mediated suppression of the human leukocyte antigen (HLA) expression [44], can only be detected in mouse models. By contrast, several immune genes, including *FCγ*, *TAL1* and *IFI16* [45], *SIGLEC-11* [46], and *SIGLEC-3* [47], have been identified exclusively in human microglia. The further identification of human-specific microglia expression profiles and resulting biological properties under physiological and pathological conditions may be implicative for future therapeutic approaches.

However, all comparative studies may be influenced by the challenging isolation of homeostatic human microglia that are commonly derived from post-mortem brains or disease-associated surgical biopsies. Under these conditions, senescence or pathology-associated cues may influence the transcriptional and functional setting of the analyzed human microglia, e.g., their transition into activated states impeding the mouse and human-model, comparability. Additionally, as adult human microglia seem to be less proliferative than their mouse homologues [48,49] and the survival of microglia from small biopsies is poor, in vitro analysis is limited to very low cell numbers that are not enough for comparable functional analysis. Importantly, it has to be taken into account that these primary cultures are not necessarily restricted to brain parenchymal-derived microglia, but may be “contaminated” by peripheral myeloid populations, possibly contributing to the above-mentioned heterogeneity.

In order to solve this problem, immortalized human microglial cell lines were established in the 1990s, including Huµglia [50], HMO6 [51], CHME5, and HMC3 [52]. However, these lines only partially reflect human microglia properties due to the loss of some antigenic characteristics critically involved in proliferation and homeostasis [53].

Increasing knowledge about the key molecules triggering microglial differentiation, maturation, and homeostatic signaling has enabled the development of hiMGL generated from hiPSC. Since 2016, a plethora of protocols have been established aiming at the in vitro generation of mature microglia-like cells mimicking their primary human counterparts concerning the expression of characteristic signature genes and functionality, including proliferation, migration, motility, integration into neural tissue, inflammatory reactions, and phagocytosis [54,55,56,57,58,59,60,61,62,63,64,65].

### 3.1. Differentiation into Microglia Progenitors In Vitro

Most of the published differentiation protocols try to mimic the in vivo developmental stages of human microglia by sequentially exposing hiPSCs to cytokines and growth factors, by adjusting the oxygen levels and/or progenitor isolation based on fluorescence-activated cell sorting (FACS) or magnetic-activated cell sorting (MACS).

**Figure 2:**
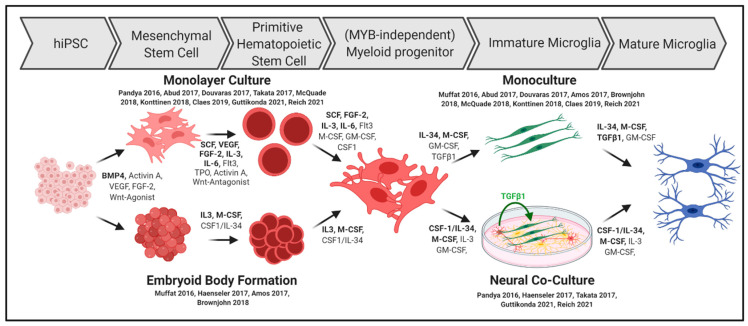
Basic differentiation strategies of iPSCs into hiMGLs indicating different culture methods and used signaling molecules, cytokines, and growth factors. The figure was created using BioRender. Available online: https://biorender.com (accessed on 17 March 2021).

The differentiation steps are summarized in Figure 2 and comprise the mesodermal differentiation of hiPSC into hemangioblasts and later into primitive hematopoietic stem cells (HPC) that are comparable to yolk sac EMPs during human microglia development [1,2].

A critical molecule inducing the mesodermal differentiation of hiPSCs is bone morphogenetic protein 4 (BMP4), which is used in nearly all protocols published. Additionally, mesodermal specification is supported by stem cell factor (SCF) or vascular endothelial growth factor (VEGF).

Wnt signaling has been implicated to affect HPC generation. While the initial differentiation of hemangioblasts is fostered by Wnt signaling [66], consecutive differentiation into primitive kinase insert domain receptor (KDR)^+^CD235^+^ hematopoetic stem cells [67] that give rise to the yolk sac progenitors (in contrast to definitive KDR^+^CD235^−^ hematopoetic stem cells) is negatively regulated by Wnt-signaling. Therefore, Wnt-agonists were initially used for mesenchymal hiPSC differentiation, but treatment was switched to Wnt-antagonists reflecting the embryonic development [60,64,68]. Interestingly, this switch must occur in a very precise time window that, surprisingly, greatly differs from 18–24 h [68], 40–48 h [64], and 6 days [60] among the protocols.

In another study, definitive hematopoiesis was induced by the application of Wnt-activator BIO and Activin-inhibitor SB431542 directly after mesodermal induction. This results in a significantly decreased number of cells expressing the triggering receptor expressed on myeloid cells 2 (TREM2), further indicating that hiMGLs can only be generated using the primitive hematopoietic stem cell lineage [56]. Using small molecules for the activation and inhibition of stage-specific pathways may be an opportunity for fine-tuning existing hiMGL differentiation protocols.

In a second step, sufficient myeloid progenitors can be generated in conventional monolayer cultures by using a time-dependent mix of cytokines, that comprises fms-like tyrosine kinase 3 (Flt3), granulocyte colony-stimulating factor (GCSF), IL-3, IL-6, SCF, VEGF, and thrombopoietin (TPO) [57,62,63]. Although their impact on mesodermal and myeloid differentiation is well characterized, individual and comprehensive implications on differentiation into hiMGLs are not well understood. However, differentiated hiMGLs that have been treated with VEGF during initial hematopoietic differentiation show enhanced CD45 and Cx3Cr1 expression, implicating the need for fine-tuning the growth factor cocktails during early differentiation [69]. However, essential markers for homeostatic functions, including Iba1, CD11b, and P2RY12, have not been influenced in this case.

In addition to growth factor and cytokine treatment, reduced oxygen levels (5% O_2_) during initial hematopoietic differentiation have also been shown to provide robust differentiation into myeloid progenitors [55,59,60,64]. Accordingly, it has been argued that O_2_ reduction may occur upon the formation of embryoid bodies (EBs) accounting for the progenitors in the inner cell mass [70]. This culture method is accompanied by cell–cell and/or cell–matrix interactions that may be beneficial for myeloid differentiation. Once the BMP4-driven mesenchymal differentiation of EBs is initiated, the growth factor and cytokine-cocktail for subsequent myeloid differentiation can be minimized to generate functional hiMGLs [56,58].

In contrast to other protocols, Muffat et al. grew iPSCs in a defined base medium chemically reflecting the cerebrospinal fluid and supplemented purely with CSF1 and IL-34, which are commonly not used until the terminal microglia differentiation. Nevertheless, EB formation spontaneously results in the formation of NPC spheroids, but also yolk sac-EBs, showing myeloid expression profiles [54].

As a result of hematopoietic and subsequent myeloid differentiation, the cells start to express CD117 and CD34 followed by CD235, CD41 [54,55,60,64], CD43 [55,59,62], and CD14 [57,63]. The time of appearance of myeloid progenitors greatly differs among protocols, ranging from 8 to 30 days, depending on the broad or frugal use of stage-specific growth factors and cytokines [58,64].

### 3.2. Final Microglia Differentiation and Maturation In Vitro

The final microglia differentiation is mainly induced by switching the cytokine cocktail to IL-34 and macrophage colony stimulating factor (MCSF) for at least 10 to 30 days, in accordance with in vivo observations of microglia survival to be dependent on the activation of colony stimulating factor 1 receptor (CSF1R) [55,58,62,63,64]. As IL-34 instead of its natural ligand colony stimulating factor (CSF1) has been shown to play a major role in microglial differentiation in mice [1,71,72], this cytokine has been used in most protocols at high concentrations (100 ng/mL), and the removal of this factor leads to depletion of the hiMGLs [58].

IL-34 and MCSF in combination lead to the expression of MERTK, ITGB5, CX3CR1, TGFBR1, IBA1, CD11b, and TMEM119, all of which are markers associated with immature and mature microglia [55]. Notably, a set of genes reported to be specific for the human microglial signature, including *MERTK*, *GPR34*, *PROS1*, *C1QA*, *GAS6*, and *P2RY12* [17], has been detected at similar or even higher levels compared to primary adult human microglia [58,59]. This expression pattern greatly differs from blood-derived monocytes (PBMC) and PBMC-derived macrophages [57]. However, most of these genes are not only expressed in hiMGLs but also in hiPSC-derived macrophages [58], indicating that such genes may not be exclusively expressed by microglia.

CSF1 instead of IL-34 can also mediate hiMGL survival in vitro [54,60], highlighting the problem of putative species differences. As murine cytokines do not effectively bind to hiMGL receptors, mice carrying the human transgenes encoding CSF1 have been generated to solve this problem. In fact, a successful hiPSC integration into the mouse tissue was dependent on the presence of human CSF1, indicating the pivotal role of CSF1R stimulation for human microglial differentiation by its “natural” ligand instead of IL-34 [73]. In addition to recombinant CSF-1 or IL-34, which seem to be critically involved in hiMGL survival [58], their cardinal role for hiMGL differentiation remains questionable due to observations of final microglial differentiation, solely supported by coculture with human astrocytes [59]. Thus, it remains to be resolved whether this could be executed by an astrocytic secretome, as a mix of various factors, including granulocyte-macrophage colony-stimulating factor (GMCSF), IL-6, IL-8, and TGFβ, that have been detected using an array for 32 human cytokines (not including IL-34 and MCSF) [74].

### 3.3. TGFβ in Microglia Differentiation and Maturation In Vitro

**Figure 3:**
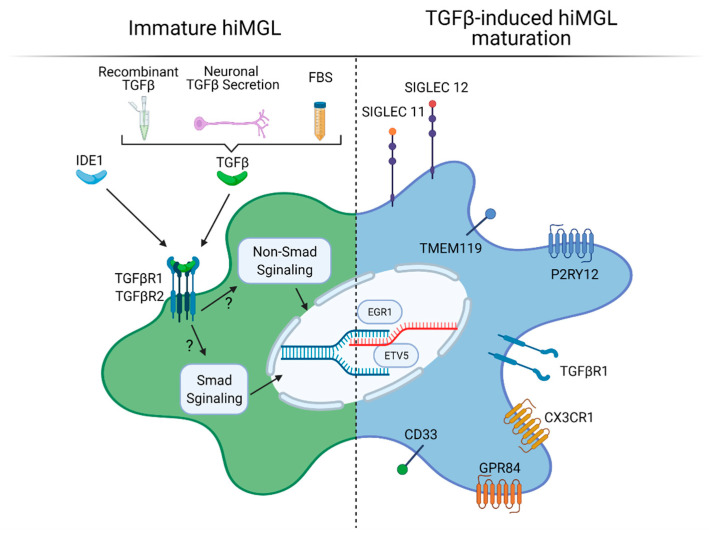
TGFβ effects on hiMGL maturation. The figure was created using BioRender. Available online: https://biorender.com (accessed on 17 March 2021).

In the developing brain, TGFβ1 has been shown to regulate rodent microglial development, homeostasis, maturation, and survival [17,24,25,75]. These actions are mediated by the TGFβ-receptor 1 (TGFBR1), which is highly expressed by both rodent microglia cultures and hiMGLs [55]. Therefore, TGFβ1 was considered as an important maturation factor for immature hiMGLs in distinct hiMGL differentiation protocols at concentrations ranging from 2 to 50 ng/mL, and its effects have been addressed in detail (Figure 3) [55,56,62,76].

The withdrawal of TGFβ from hiMGL medium reduces the expression of surface receptors encoding genes *P2RY12*, *TGFβR1*, *CX3CR1*, *GPR84*, and *CD33*, as well as microglial transcription factors *EGR1* and *ETV5* [55]. In total, 24h after TGFβ treatment, more than 2000 genes were found to be differentially expressed [55]. Interestingly, many of these genes are associated with neurodegenerative diseases. The removal of TGFβ leads to significant changes that have been identified as Alzheimer’s disease (AD) GWAS loci genes, including *TREM2* and *APOE*, indicating the relevance of TGFβ for microglial homeostasis and maintenance [55]. Based on these data, pathways regulating mitosis and proliferation are also modulated by TGFβ treatment [55] similar to observations in mice [75]. Finally, the differentiation and proliferation of already differentiating hiMGLs are significantly diminished when TGFβ is withdrawn in vitro [62].

Intriguingly, TGFβ actions can be mimicked by incubation with a more stable small molecule, “Inducer of Definitive Endoderm1” (IDE1), mediating the phosphorylation of TGFβ downstream signaling [77]. It is noteworthy that the substitution of TGFβ by IDE1 results in similar growth kinetics compared with TGFβ treatment and higher IDE1 concentrations, further increasing cell proliferation. Correlation analysis demonstrates that gene expression of IDE1-treated iMGLs cluster with TGFβ-treated iMGLs and even more with fetal and adult microglia [62].

Although TGFβ has not been added as a recombinant cytokine in all hiMGL protocols, the use of 10% fetal bovine serum (FBS) supplemented medium in distinct differentiation protocols might contain 1–2 ng/mL of TGFβ [78], supporting the microglia maturation process [64,68]. In this context, the further development of immature hiMGLs could benefit from these “hidden” TGFβ effects, and its well-described functions to regulate microglial activation might additionally play important roles [79].

### 3.4. Cell–Cell and Cell–Matrix Interactions in hiMGL Differentiation and Maturation In Vitro

Since even fine-tuned culture media do not fully reproduce the complex microglial niche in the developing three-dimensional brain, neuronal and astrocyte-derived signals have been hypothesized to influence microglial identity and may also be crucial for the generation of fully maturated hiMGLs. Principle component analysis of the transcriptome reveals that hiMGLs exposed to hiPSC-derived mature neuron-conditioned medium slightly shift their transcriptome to fetal microglia [54]. Direct co-culture with neurons results in the expression of human-specific sialic acid-binding immunoglobulin-type lectin (*SIGLEC) 11* and *SIGLEC 12* that interact with the neuronal glycocalyx, suppressing inflammation, and thus, may maintain microglia in a homeostatic state [55]. Additionally, the co-culture of neurons and hiMGLs results in a more dynamically ramified morphology and enhanced migration abilities, as well as an overall reduced secretion of chemokines and cytokines, indicating mechanisms of neuron-mediated homeostasis [58]. A recent study comparing neuron-astrocyte co-culture vs. monocultured iMGLs shows that microglial signature gene expression is barely different among these groups, indicating that both approaches can lead to fully differentiated microglia [76]. However, genes related to so-called “disease-associated microglia” (DAM) have been upregulated in the monoculture group, including *TREM2* that is a “risk gene” for neurodegenerative diseases. This indicates that cell–cell interactions are not necessary for the differentiation and maturation of iMGLs, but for adopting a homeostatic in vivo-like state.

It has been hypothesized that the exposure of neural progenitor cell (NPC)-conditioned medium to developing hiMGLs might mimic the environmental cues as microglia develop in synchrony with neurons [65]. In fact, hiMGL maturation with NPC-conditioned medium results in the higher expression of *TMEM119* compared with unconditioned media or astrocyte and oligodendrocyte precursor-conditioned media.

Although not further investigated, TGFβ signaling may be partly responsible for these effects, as this factor is continuously secreted by developing and adult neural tissue [18,23,80]. However, the molecular mechanisms of direct and indirect interactions of hiPSCs with neural cells boosting their differentiation and maturation for in vitro applications remain to be studied in more detail.

Additional factors affecting iMGL differentiation and maturation have been analyzed in single studies, i.e., addressing the potential of the extracellular matrix. In fact, fibronectin coating results in a more ramified morphology compared to poly-D-lsine, collagen I, gelatin, and laminin coating. Furthermore, additional treatment with (soluble) CD200 and chemokine (C-X3-C motif) ligand 1 (CX3CL1) in combination did only result in minor changes of microglia signature genes, indicating that these factors influence microglial functions rather than differentiation or maturation [55,76].

## 4. Summary and Conclusions

Taken together, the generation of hiMGLs from hiPSC closely resembles the well-described microglia differentiation and maturation processes observed in mice and thus might represent a powerful tool for studying the functional aspects of human microglia. However, the prerequisite for such studies using hiMGLs is that these in vitro generated cells share the full molecular signatures as their in vivo counterparts. To overcome the limitations of human in vivo studies, comparative analysis of hiMGLs and mouse-derived iMGLs should be performed, examining common and divergent maturation pathways.

For a better understanding of specific developmental and maturation mechanisms, the molecular crosstalk of (how certain known and unknown) homeostatic and maturation modulators guiding the specification of microglial phenotypes at certain developmental stages and in different brain regions in mice should be dissected in more detail. The subsequent translation of these investigations to differentiation protocols will pave the way for a reliable and more robust in vitro generation of microglia sharing the typical expression signature, as well as functionality with their in vivo counterparts. This will also be a prerequisite for the deeper understanding of the molecular background of (human) microglia, including their putative contribution to neurodegenerative diseases. This point will be even more relevant for the possibility to employ hiMGLs for future microglia replacement strategies.

In particular, recent achievements underlining the importance of TGFβ1 and microglial TGFβ signaling need to be considered in hiMGL differentiation protocols, and further studies addressing the specific effects of TGFβ1 in human microglia and hiMGLs have to be performed in order to elucidate the molecular mechanisms underlying TGFβ1-mediated effects. Moreover, the benefit of TGFβ1 as an additional factor for hiMGL maturation has to be analyzed and validated in more detail in order to generate hiMGLs that share essential features of human microglia in vivo.

## Data Availability

Not Applicable.

## Abbreviations

AD	Alzheimer’s disease
BAM	border-associated macrophages
BBB	blood brain barrier
BMP4	bone morphogenetic factor 4
CNS	central nervous system
CSF1R	colony stimulating factor-1 receptor
CX3CL1	chemokine (C-X3-C motif) ligand 1
EB	embryoid body
EMP	erythromyeloid progenitors
FACS	fluorescence-activated cell sorting
Flt3	fms-like tyrosine kinase 3
GCSF	granulocyte colony-stimulating factor
GMCSF	granulocyte-macrophage colony-stimulating factor
hiMGL	human-induced microglia-like cells
hiPSC	human-induced pluripotent stem cells
HPC	hematopoietic stem cells
IDE	inducer of definitive endoderm
IL	interleukin
KDR	kinase insert domain receptor
MACS	magnetic-activated cell sorting
PBMC	peripheral Blood Mononuclear Cell
SCF	stem cell factor
SIGLEC	Sialic acid-binding immunoglobulin-type lectin
TGFBR1	transforming growth factor β receptor 1
TGFβ	transforming Growth Factor beta
TPO	thrombopoietin
